# Draft Genome Sequence of *Cystobacter ferrugineus* Strain Cbfe23

**DOI:** 10.1128/genomeA.01601-16

**Published:** 2017-02-09

**Authors:** Shukria Akbar, Scot E. Dowd, D. Cole Stevens

**Affiliations:** a Department of Biomolecular Sciences, University of Mississippi, University, Mississippi, USA; b MR DNA, Molecular Research LP, Shallowater, Texas, USA

## Abstract

In an effort to explore myxobacterial natural product biosynthetic pathways, the draft genome sequence of *Cystobacter ferrugineus* strain Cbfe23 has been obtained. Analysis of the genome using antiSMASH suggests a multitude of unique natural product biosynthetic pathways. This genome will contribute to the investigation of secondary metabolism in other myxobacterial species.

## GENOME ANNOUNCEMENT

Myxobacteria are a prolific source of natural products with members of the genus *Cystobacter* producing a variety of structurally diverse antibiotics (1–6). Isolated from a soil sample in China collected in 1982, *Cystobacter ferrugineus* strain Cbfe23, DSM 52764, was recently reported to produce the novel diterpene cystodienoic acid (7). Here, we report a draft genome sequence for *C. ferrugineus* strain Cbfe23 collected in an effort to explore myxobacterial natural products.

*C. ferrugineus* was acquired from the German Collection of Microorganisms (DSM) in Braunschweig (DSM 52764) and cultivated using the suggested medias and conditions. Genomic DNA was isolated using a GeneJET genomic DNA purification kit (ThermoFisher). Sequencing was performed at MR DNA (Shallowater, TX) using an Illumina HiSeq system. The libraries were prepared using a Nextera DNA sample preparation kit (Illumina) following the manufacturer’s user guide. Following the library preparation, the final concentration of the library (13.0 ng/μL) was measured using the Qubit dsDNA HS assay kit (Life Technologies), and the average library size (845 bp) was determined using the Agilent 2100 Bioanalyzer (Agilent Technologies). The libraries were pooled and diluted (to 10.0 pM) and sequenced paired end for 500 cycles with an average coverage of 50×. An initial annotation was completed using the Rapid Annotations using Subsystems Technology (RAST) server (8) with further annotation requested by the NCBI Prokaryotic Genome Annotation Pipeline (9, 10). The draft genome contains 12,051,756 bp with 74 identified RNAs, 9,992 coding sequences, and a 68.5% G+C content across 42 contigs containing protein-encoding genes.

Using antiSMASH version 3.0.5, 44 unique secondary metabolite biosynthetic pathways were identified including pathways for 10 terpenes, eight nonribosomal peptides, seven bacteriocins, four lantipeptides, four polyketides, three hybrid nonribosomal peptide-polyketides, and three microcins (11). Sequence homology between the identified pathways and reported myxobacterial biosynthetic pathways suggests that *C. ferrugineus* may produce metabolites structurally similar to tubulysin, myxochelin, and cystobactamid natural products (3, 12, 13). We believe the draft genome sequence will help facilitate characterization of myxobacterial secondary metabolism.

### Accession number(s).

This whole-genome shotgun project has been deposited in DDBJ/ENA/GenBank under the accession number MPIN00000000. The version described in this paper is the first version, MPIN00000000.1.

## References

[1] Schäberle TF, Lohr F, Schmitz A, König GM 2014 Antibiotics from myxobacteria. Nat Prod Rep 31:953–972. doi:10.1039/c4np00011k. 24841474

[2] Wenzel SC, Müller R 2009 The impact of genomics on the exploitation of the myxobacterial secondary metabolome. Nat Prod Rep 26:1385–1407. doi:10.1039/b817073h. 19844638

[3] Baumann S, Herrmann J, Raju R, Steinmetz H, Mohr KI, Hüttel S, Harmrolfs K, Stadler M, Müller R 2014 Cystobactamids: myxobacterial topoisomerase inhibitors exhibiting potent antibacterial activity. Angew Chem Int Ed Engl 53:14605–14609. doi:10.1002/anie.201409964. 25510965

[4] Feng Z, Qi J, Tsuge T, Oba Y, Kobayashi T, Suzuki Y, Sakagami Y, Ojika M 2005 Construction of a bacterial artificial chromosome library for a myxobacterium of the genus *Cystobacter* and characterization of an antibiotic biosynthetic gene cluster. Biosci Biotechnol Biochem 69:1372–1380. doi:10.1271/bbb.69.1372. 16041144

[5] Cortina NS, Revermann O, Krug D, Müller R 2011 Identification and characterization of the althiomycin biosynthetic gene cluster in *Myxococcus xanthus* DK897. Chembiochem 12:1411–1416. doi:10.1002/cbic.201100154. 21626639

[6] Zander W, Gerth K, Mohr KI, Kessler W, Jansen R, Müller R 2011 Roimatacene: an antibiotic against Gram-negative bacteria isolated from *Cystobacter ferrugineus* Cb G35 (myxobacteria). Chemistry 17:7875–7881. doi:10.1002/chem.201003677. 21618624

[7] Raju R, Mohr KI, Bernecker S, Herrmann J, Müller R 2015 Cystodienoic acid: a new diterpene isolated from the myxobacterium *Cystobacter* sp. J Antibiot (Tokyo) 68:473–475. doi:10.1038/ja.2015.8. 25690355

[8] Overbeek R, Olson R, Pusch GD, Olsen GJ, Davis JJ, Disz T, Edwards RA, Gerdes S, Parrello B, Shukla M, Vonstein V, Wattam AR, Xia F, Stevens R 2014 The SEED and the Rapid Annotation of microbial genomes using Subsystems Technology (RAST). Nucleic Acids Res 42:D206–D214. doi:10.1093/nar/gkt1226. 24293654 PMC3965101

[9] Angiuoli SV, Gussman A, Klimke W, Cochrane G, Field D, Garrity G, Kodira CD, Kyrpides N, Madupu R, Markowitz V, Tatusova T, Thomson N, White O 2008 Toward an online repository of standard operating procedures (SOPs) for (meta)genomic annotation. Omics 12:137–141. doi:10.1089/omi.2008.0017. 18416670 PMC3196215

[10] Tatusova T, DiCuccio M, Badretdin A, Chetvernin W, Ciufo S, Li W 2013 Prokaryotic genome annotation pipeline. *In* The NCBI handbook, 2nd ed. National Center for Biotechnology Information, Bethesda, MD https://www.ncbi.nlm.nih.gov/books/NBK174280/.

[11] Weber T, Blin K, Duddela S, Krug D, Kim HU, Bruccoleri R, Lee SY, Fischbach MA, Müller R, Wohlleben W, Breitling R, Takano E, Medema MH 2015 antiSMASH 3.0-a comprehensive resource for the genome mining of biosynthetic gene clusters. Nucleic Acids Res 43:W237–W243. doi:10.1093/nar/gkv437. 25948579 PMC4489286

[12] Chai Y, Shan S, Weissman KJ, Hu S, Zhang Y, Müller R 2012 Heterologous expression and genetic engineering of the tubulysin biosynthetic gene cluster using Red/ET recombineering and inactivation mutagenesis. Chem Biol 19:361–371. doi:10.1016/j.chembiol.2012.01.007. 22444591

[13] Li Y, Weissman KJ, Müller R 2008 Myxochelin biosynthesis: direct evidence for two- and four-electron reduction of a carrier protein-bound thioester. J Am Chem Soc 130:7554–7555. doi:10.1021/ja8025278. 18498160

